# Natural selection contributes to the myopia epidemic

**DOI:** 10.1093/nsr/nwaa175

**Published:** 2020-08-17

**Authors:** Erping Long, Jianzhi Zhang

**Affiliations:** Department of Ecology and Evolutionary Biology, University of Michigan, Ann Arbor, MI 48109, USA; State Key Laboratory of Ophthalmology, Zhongshan Ophthalmic Center, Sun Yat-sen University, Guangzhou 510060, China; Department of Ecology and Evolutionary Biology, University of Michigan, Ann Arbor, MI 48109, USA

**Keywords:** allele frequency, natural selection, antagonistic pleiotropy, evolution, myopia, reproduction

## Abstract

The prevalence of myopia, or nearsightedness, has skyrocketed in the past few decades, creating a public health crisis that is commonly attributed to lifestyle changes. Here we report an overall increase in the frequencies of myopia-associated mutant alleles over 25 years among participants of the UK Biobank. Although myopia itself appears to be selected against, many of the mutant alleles are associated with reproductive benefits, suggesting that reproduction-related selection inadvertently contributes to the myopia epidemic. We estimate that, in the UK alone, natural selection adds more than 100 000 myopia cases per generation, and argue that antagonistic pleiotropy be broadly considered in explaining the spreads of apparently disadvantageous phenotypes in humans and beyond.

## Teaser

Analysis of the UK Biobank shows that the rapid increase in the prevalence of myopia is in part attributable to positive selection for myopia-associated risk alleles due to antagonistic pleiotropy.

## INTRODUCTION

A number of human diseases have risen in prevalence in the last few decades [1]. Although the exact causes are often unknown, several non-mutually exclusive explanations exist. First, the increased prevalence could result from improved medical diagnosis. Second, it could be a consequence of demographic changes [2,3]. For instance, many diseases are age-dependent such that a change in the population age structure could alter disease prevalence. Third, it could be caused by environmental changes [4]. Fourth, it could be due to increases in the frequencies of mutant alleles causing the disease, either because the disease phenotype actually increases fitness [5] or because these alleles have other phenotypic effects that are advantageous (i.e. antagonistic pleiotropy) [6].

Myopia is the most common cause of distant visual impairment. The prevalence of myopia has approximately doubled in the past three decades, and it is predicted that 49.8% of the world population, or 4.9 billion people, will develop myopia in 2050 [7]. The global potential productivity loss due to uncorrected myopia amounts to 244 billion US dollars per year [8], and the global costs of facility and personnel for establishing refractive care services are 20 billion dollars per year [9]. Environmental risk factors such as near-work intensity and lack of outdoor activity are thought to play major roles in the myopia epidemic [10], whereas the contribution of genetics is unclear. Given that genome-wide association studies (GWAS) have identified a large number of genetic variants associated with myopia [11–14], we are interested in the possibility that the rapid rise in myopia prevalence is at least in part attributable to natural selection for myopia-associated alleles. Specifically, we ask the following three questions. Are myopia-associated alleles under natural selection? If so, is the selection acting on the myopia phenotype or some other phenotypes? What is the quantitative impact of the selection on myopia prevalence?

## RESULTS AND DISCUSSION

### Myopia prevalence increased over six birth cohorts of UK Biobank participants

**Figure 1.**
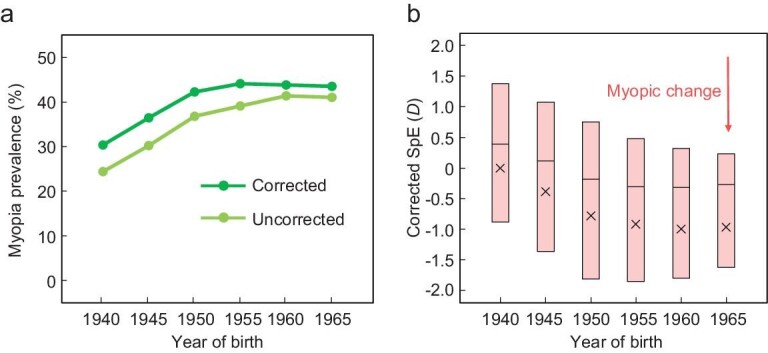
Prevalence of myopia increased over birth cohorts in UK Biobank. (a) Myopia prevalence rose over six five-year birth cohorts. The light green curve shows the uncorrected prevalence, whereas the dark green curve shows the adjusted prevalence (at age 40) after the correction for the hyperopic effect of aging. The X-axis shows the starting year of each birth cohort. The 95% confidential interval is too small to be visible. (b) Corrected spherical equivalent (SpE) measured in diopter (*D*) across the six birth cohorts. The cross symbol represents the mean SpE, the band shows the median and the box indicates the middle 50% of individuals.

To address these questions, we analyzed a large number of individuals with genotype and phenotype data in the UK Biobank [15]. We excluded individuals with invalid or unreliable refractive records, non-European ancestry, or genetic kinship to others in the Biobank, to reach the study set of 63 185 unrelated individuals of European ancestry born between 1940 and 1969 (see Methods). Dividing these individuals into six five-year birth cohorts (1940–1944, 1945–1949, …, and 1965–1969), we found that the myopia prevalence increased from 24.4% to 41.0% over these cohorts (light green line in Fig. 1a). Because all subjects were phenotyped between 2006 and 2010, which correspond to different ages for different cohorts, we corrected the hyperopic effect of aging on myopia [16] (see Methods) and estimated that the myopia prevalence at age 40 rose from 30.3% to 43.5% over 25 years (dark green line in Fig. 1a), similar to the finding of Williams *et al.*’s meta-analysis across multiple European populations [17]. Spherical equivalent (SpE), a measure of myopia severity, similarly showed a myopic shift over this period, both before (Fig. S1) and after (Fig. 1b) the correction for the hyperopic effect of aging.

### Positive selection for myopia risk alleles

**Figure 2.**
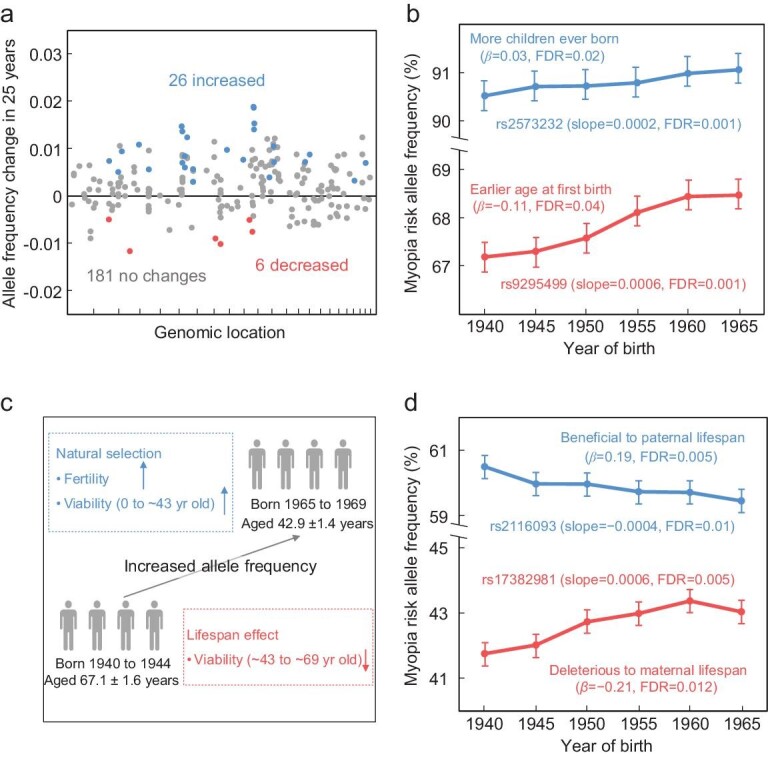
More myopia-associated alleles increased than decreased in frequency over birth cohorts in UK Biobank. (a) Changes in the frequencies of myopia-associated risk alleles over 25 years. Each dot represents one SNP, whose genomic coordinate is shown on the X-axis (chromosome 1 to 22 for each interval from left to right). Blue and red dots indicate SNPs with significant frequency changes, whereas gray dots indicate those without significant changes. The horizontal line indicates no frequency change. (b) Two examples of significant frequency increases of myopia-associated risk alleles over the six birth cohorts. These alleles are subsequently found to be significantly associated with the age at first birth (AFB) or number of children ever born (NEB). Error bars show 95% confidence intervals. β, effect size on AFB or NEB per allele. Slope refers to the allele frequency change per year based on a linear regression. (c) Potential causes of an allele frequency increase over the birth cohorts. An upward arrow indicates a positive effect of the focal allele or a linked allele, whereas a downward arrow indicates a negative effect. Birth cohort ages shown are mean ± standard deviation. (d) Two myopia-associated risk alleles with significant allele frequency changes over the six birth cohorts are associated with parental lifespans. β, effect size on lifespan per allele. Slope refers to the allele frequency change per year based on a linear regression.

The Biobank genotype data included 471 single nucleotide polymorphisms (SNPs) that had been reported to be associated with myopia-related traits in other samples, 213 of which remained after we verified their significant associations with myopia in our sample and excluded those with high linkage disequilibrium (see Methods). In the above verification, age, sex and the first 10 genetic principal components provided by the Biobank were used as covariates to guard against spurious associations. Among the 213 verified SNPs, 32 exhibited significant allele frequency changes over the six birth cohorts (false discovery rate or FDR < 0.05 from linear regression; see Methods). Interestingly, more myopia risk alleles rose than declined in frequencies (26 rose *vs.* 6 declined, *P* = 0.00054, two-tailed binomial test; Fig. 2a). The speeds of allele frequency changes are quite high for the 32 SNPs that had significant allele frequency changes over the birth cohorts, especially those with increased frequencies (Fig. 2a). For example, the frequency of the myopia risk allele rose at a speed of 0.0002 and 0.0006 per year at SNPs rs2573232 and rs9295499, respectively (Fig. 2b).

As a negative control, we generated 100 random sets of 213 SNPs with matched allele frequencies (±1%) and recombination rates (±0.05 cM/Mb) from genome-wide variants in the Biobank. But none of these sets contained ≥32 SNPs with significantly altered allele frequencies over the six birth cohorts. There is also no significant bias in allele frequency increase or decrease in these sets (on average, six significantly increased vs. five significantly decreased SNPs per set). One difference between this negative control and the analysis of myopia-associated SNPs is that we had removed the SNP with the relatively high *P*-value when two myopia-associated SNPs were in high linkage disequilibrium. We found that our results would have remained qualitatively unchanged had we randomly removed one of the two SNPs. Thus, the detected overall increase of the frequencies of myopia risk alleles is genuine.

At the time of their Biobank participation, the age of the earliest birth cohort considered was 67.1 ± 1.6 (mean ± SD) years while that of the latest cohort was 42.9 ± 1.4 years. Hence, an allele frequency increase detected above could be due to direct (or linked) positive Darwinian selection for the allele because it (or a linked allele) enhances viability before the age of ∼43 and/or reproduction (Fig. 2c). Alternatively, the allele frequency increase over the birth cohorts could be explained if the allele (or a linked allele) lowers the survivorship from the age of ∼43 to ∼67 (i.e. lifespan reduction) [18] (Fig. 2c). To investigate the latter possibility, we tested the associations between the 213 verified myopia-associated SNPs and the lifespans of the parents of Biobank participants (see Methods). Twenty-one SNPs showed significant associations with the lifespan of one or both parents (FDR < 0.05). Among them, two SNPs had significantly altered allele frequencies over the six birth cohorts; in both cases, the direction of the allele frequency alteration is explainable by the direction of the lifespan association (Fig. 2d). After the exclusion of these two SNPs, myopia risk allele frequencies increased at 25 SNPs but decreased at only five SNPs over the six birth cohorts (Table S1), revealing an overall spread of myopia risk alleles driven by positive selection (*P* = 0.00032, two-tailed binomial test) for these alleles or linked alleles.

The positive selections responsible for the frequency increases of the 25 myopia risk alleles are strong. For example, for the aforementioned SNPs rs2573232 and rs9295499 (Fig. 2b), we estimated that the selective advantages of the risk (or linked) alleles are respectively 0.006 and 0.022 per generation, under the assumption that the risk (or linked) alleles are completely dominant over the corresponding protective alleles in terms of fitness and that the generation time is 25 years (see Methods). In evolutionary terms, these selection coefficients are huge. For comparison, the coefficient of selection for the classic human glucose-6-phosphate dehydrogenase (G6PD) deficiency allele that lowers the risk of malaria is 0.02–0.05 in a region endemic for malaria [19].

### Myopia is selected against

**Figure 3.**
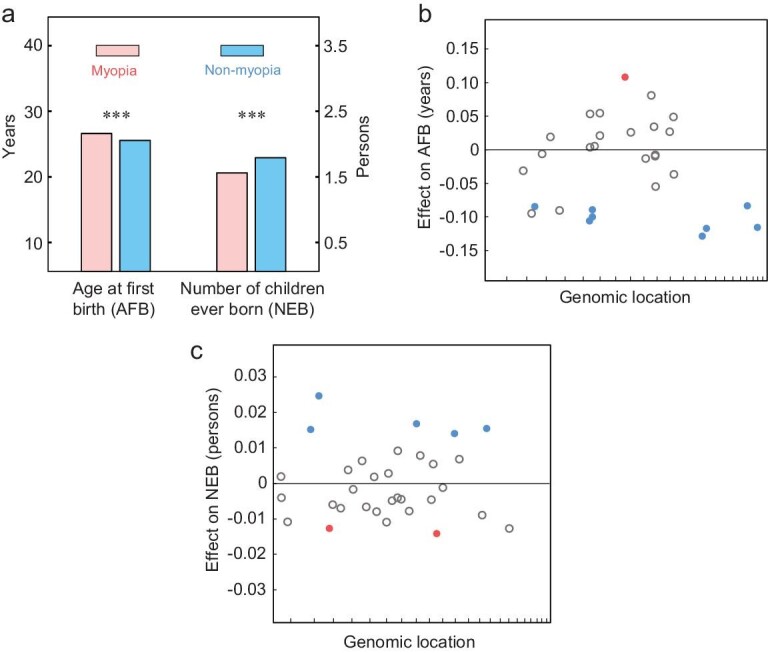
Many myopia-associated alleles subject to natural selection are also associated with reproduction. (a) Myopic people have significantly greater mean AFB and smaller mean NEB than non-myopic people. The error bars showing the standard error are too small to be visible. ***, *P* < 0.001, two-tailed *t*-test. (b and c), Effects on AFB (b) and NEB (c) of myopia-associated risk alleles that have significant frequency changes over the six birth cohorts. Each circle represents an SNP, whose genomic coordinate is shown on the X-axis (chromosome 1 to 22 for each interval from left to right). The horizontal line indicates zero effect. Significant effects (FDR < 0.05) are shown by solid circles, whereas non-significant effects are indicated by open gray circles. Among solid circles, blue and red respectively indicate allele frequency increases and decreases.

Is the positive selection for myopia-associated alleles caused by a potential benefit of the myopia phenotype? To answer this question, we compared between myopic and non-myopic individuals in their reproductive traits, including *a*ge at *f*irst live *b*irth (AFB) and *n*umber of children ever *b*orn (NEB). Owing to improvements in hygiene and reduction in prenatal, infant and child mortality in industrialized societies, AFB and NEB have emerged as the gold standard in measuring lifetime reproductive success [20]. We found that myopia is associated with delayed reproduction (*P *= 2.5 × 10^−55^) and fewer offspring (*P *= 8.2 × 10^−110^) (Fig. 3a). These observations are consistent with the previous finding that myopia is highly positively correlated with educational attainment [21], which is in turn associated with delayed reproduction and fewer offspring [22]. Thus, the myopia phenotype itself is actually selected against, presumably indirectly.

### Positively selected myopia risk alleles are associated with reproductive advantages

We then tested the associations between the 30 myopia-associated SNPs subject to (direct or linked) natural selection and the above reproductive traits at an FDR of 5% (Table S1). Again, age, sex and the first 10 genetic principal components provided by the Biobank were used as covariates to guard against spurious associations (see Methods). Of the 25 positively selected myopia risk alleles, 12 are significantly associated with at least one of these traits, including eight alleles reducing AFB (Fig. 3b) and five alleles increasing NEB (Fig. 3c) with one overlap. Of the five negatively selected myopia risk alleles, three are significantly associated with at least one of these traits, including one increasing AFB (Fig. 3b) and two reducing NEB (Fig. 3c). In all of these significant associations, the direction of the selection on an allele inferred from allele frequency changes over the six birth cohorts is explainable by the direction of the allele's association with reproduction (*P* < 10^−4^, binomial test). Furthermore, for both reproductive traits, the selection coefficient estimated from allele frequency changes for an SNP correlates significantly with the allele's reproductive effect size among SNPs with significant reproductive effects (AFB: Pearson's *r* = −0.67, *n* = 9, *P* = 0.049; NEB: *r* = 0.84, *n* = 7, *P* = 0.017). The results in Fig. 3b and c generally hold when myopic and non-myopic individuals are separately examined (Table S1). As a negative control, we randomly picked 30 SNPs with matched allele frequencies and recombination rates, but found only three SNPs associated with at least one of the reproductive traits, a significantly lower proportion than that in the actual data (*X*^2 ^= 11.4, *P *= 0.0007). That the myopia-associated SNPs are also associated with reproduction means that these SNPs have statistical pleiotropy [23]. Although statistical pleiotropy may not represent biological pleiotropy where one mutation causally affects multiple traits [24], the impact of pleiotropy on allele frequency changes is similar because of linked selection. Thus, the positive selection on many myopia risk alleles is probably owing to their (statistically) pleiotropic effects on reproductive success. It is possible that the 13 positively selected myopia risk alleles that are not significantly associated with reproductive success are associated with viability prior to reproduction, but this hypothesis cannot be tested using the Biobank data because of the lack of participants before the age of 40.

### Quantitative impact of the selection on myopia prevalence

**Figure 4.**
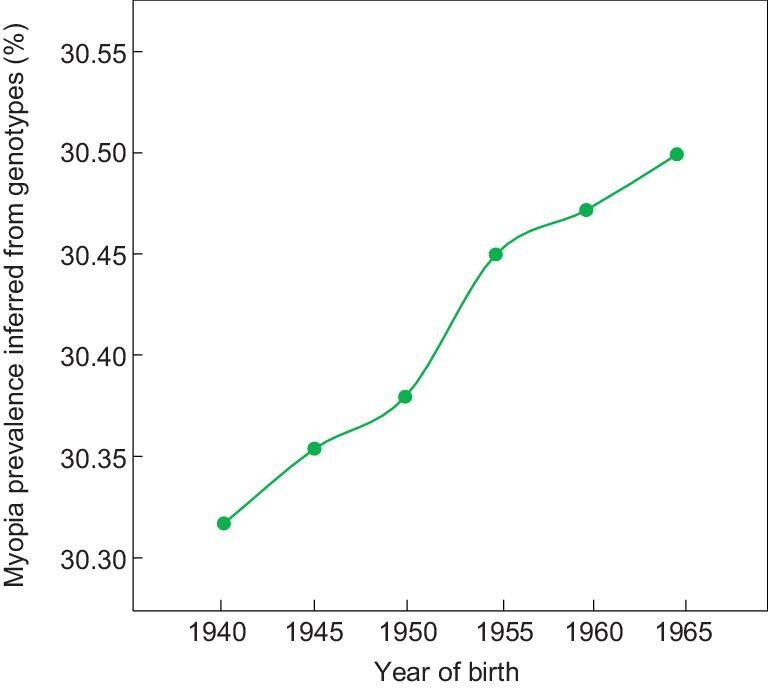
Impact of the positive selection on myopia prevalence. Shown are mean myopia prevalence inferred from genotypes of each birth cohort. Standard errors are too small to be visible.

To assess the impact of the natural selection and resulting genetic changes on myopia prevalence, we predicted changes in myopia prevalence from genotype changes using GCTA-Simu under an additive genetic model (see Methods). The prevalence of myopia at age 40 was set at 30.3% for the birth cohort of 1940–1944, as aforementioned. Because the SNP heritability of myopia was reported to be between 35% and 40% [25,26], to be conservative, we used a heritability of 35% in our simulation. We found that, in 25 years, the genetic changes at 192 myopia-associated SNPs (upon the removal of 21 with lifespan effects) added a 0.211 percentage point to myopia prevalence (Fig. 4), which equals 1.6% of the overall increase in myopia prevalence during the same time. Thus, our finding does not alter the prevailing view about the importance of environmental factors in the myopia epidemic. Nonetheless, based on the UK population of 55 429 643 in 1969 [27], the positive selection adds 116 957 myopia cases per generation in the UK alone, indicating that the selection has a substantial impact on the number of myopia cases. East Asia has the most serious myopia epidemic among all regions [10], so future replication of the present study in East Asian populations would be especially relevant.

### Robustness of the positive selection signal

Recent studies showed that population stratification could lead to errors in GWAS-based inference of natural selection, so using relatively homogeneous samples such as the UK Biobank data is preferred [28,29]. As mentioned, we have taken steps to further reduce potential errors in the use of the Biobank data by excluding individuals with non-European ancestry or with genetic kinship to others in the Biobank from all analyses and by using age, sex and the first 10 genetic principal components as covariates in association studies. In addition, negative controls were used in the analysis of allele frequency changes and that of association with reproductive traits.

To further minimize the impact of potential geographic variations in genotypes and phenotypes and technical biases, we performed a new test of association by including the assessment center where participants registered with the Biobank, the genotyping batch, and the local ancestry surrounding the SNP being tested (see Methods) as additional covariates. This analysis verified 189 significant associations from the 471 SNPs previously reported to be associated with myopia-related traits in other samples. Among these significant SNPs, 24 exhibited significant allele frequency changes over the six birth cohorts and more myopia risk alleles rose than declined in frequencies (20 increased vs. 4 decreased, *P* = 0.0015, two-tailed binomial test; Fig. S2).

To prevent potential contaminations of signals between the analysis of genetic association and that of allele frequency changes when the same sample is used, we considered all 471 SNPs that were previously reported to be associated with myopia-related traits in other samples. A total of 298 SNPs remained after the exclusion of those with strong linkage disequilibrium. Among these 298 SNPs, 41 exhibited significant allele frequency changes over the six birth cohorts; 34 increased while only 7 decreased in frequencies (*P* = 0.000026, two-tailed binomial test; Fig. S3).

Together, the steps taken in our main analyses and the above additional analyses suggest that our results are genuine and are not due to potential confounding factors.

### Implications

In summary, we provided evidence that positive selection has contributed to the rise of myopia prevalence in the UK. Nevertheless, this selection is not because myopia itself is beneficial, but is likely because many myopia-associated risk alleles are also associated with reproductive success. That these positively selected alleles are still unfixed despite having substantial selective advantages suggests the possibility that their (direct or linked) fitness advantages had not been realized until recently, probably as a result of environmental changes. The underlying mechanism of the antagonistic pleiotropy of myopia risk alleles is currently unknown, but such statistical pleiotropy is prevalent in humans [23]. For instance, alleles associated with increased educational attainment are also associated with decreased reproductive success even after the control of the educational level [22]. Furthermore, genetic manipulations of model organisms found widespread biological antagonistic pleiotropy [30]. Hence, we suggest that biological or statistical antagonistic pleiotropy be considered as a potential cause in the study of other human diseases such as cancer and type 2 diabetes that are quickly rising in prevalence [1].

World War II (WWII) may be an additional, temporary factor in the rise of myopia prevalence observed in the Biobank. Presumably, myopic adults had reduced probabilities to be drafted and hence were spared from the tremendous British military casualties in WWII that amounted to nearly 1% of the total UK population. Due to the heritability of myopia, there could be a short-term surge of myopia prevalence among the generation born during or after WWII. Indeed, the increase in myopia prevalence was greater for the birth cohorts covering 1945–1954 than later cohorts (Fig. 1a). Nevertheless, genotype-based prediction of myopia prevalence does not show a higher rate of increase in early than late cohorts (Fig. 4), suggesting that WWII was unlikely a major driver of the increases of myopia-associated risk alleles. Comparison between the UK data and those from European countries with comparatively lower military casualties in WWII would be required to rigorously test the war effect on the myopia prevalence.

## METHODS

### Study participants and data used

The UK Biobank data comprise ∼0.5 million participants aged from 40 to 70 years, recruited between 2006 and 2010 in 22 assessment centers throughout the UK, and followed up for a variety of health conditions from their recruitment date until 17 February 2016 or their date of death [15]. Informed consent was obtained from all participants [15]. Participants provided a blood sample, from which DNA was extracted and genotyped using the UK BiLEVE Axiom array or Affymetrix Axiom array [15]. We used the imputed genotypes available from the UK Biobank; full details can be found in the official UK Biobank imputation document (http://biobank.ctsu.ox.ac.uk/crystal/crystal/docs/impute_ukb_v1.pdf, accessed on 23 April 2019). The current study was approved by UK Biobank (reference no. 48678), and the analyses presented were based on data from 488 377 individuals accessed through the UK Biobank (http://www.ukbiobank.ac.uk) on 23 April 2019.

The ophthalmic examination of refractive status was conducted using non-cycloplegic autorefraction (Tomey RC5000; Tomey GmbH Europe, Erlangen-Tennenlohe, Germany) [31]. SpE was calculated as the sphere power plus half the cylinder power, and the mean SpE of two eyes of each individual was used in subsequent analysis. The presence of myopia was defined by SpE < −0.5 diopters. Given that the adult refractive error changes over time, we used the five-year change in SpE determined from the Beaver Dam Eye (longitudinal) Study of populations of European ancestries to correct the hyperopic effect of aging from 40 to 70 years [16]. Specifically, using this effect and the age of the person at the eye exam, we predicted each individual's refractive error at the age of 40.

From the entire set of 488 377 individuals with genotype information, we removed individuals with any of the following conditions: refractive error not measured or not reliably measured, cataract surgery history, refractive surgery history, cornea graft surgery, eye surgery within four weeks, self-report of non-white British ethnicity, genetic principal components indicative of non-European ancestry, at least one relative in the data identified by genetic kindship, and outlying level of genetic heterozygosity. The remaining individuals were divided into six five-year birth cohorts based on the birth year: 1940–1944, 1945–1949, 1950–1954, 1955–1959, 1960–1964 and 1965–1969. Individuals of following birth years were excluded because of the small sample sizes (*n* < 100): 1934 (*n* = 1), 1935 (0), 1936 (4), 1937 (30), 1938 (57), 1939 (92), 1970 (21) and 1971 (1). Our final sample of unrelated individuals of European ancestry born between 1940 and 1969 included 63,185 individuals.

### SNPs associated with myopia

The NHGRI GWAS Catalog [32] was downloaded from www.ebi.ac.uk/gwas (assessed 16 April 2019). We focused on 471 SNPs known to be associated with myopia, refractive error, SpE, severe or pathological myopia or age diagnosis of myopia. Because these SNPs may not be associated with myopia among Biobank participants, we used quantitative association analysis of PLINK v2 [33] to verify the associations of these SNPs with the refractive error of Biobank participants, while age, sex and the first 10 genetic principal components provided by the Biobank were fitted as covariates to avoid spurious associations. SNPs with minor allele count <5, missing genotype rate >0.05 or imputation information score <0.3 were excluded. A genetic relationship matrix was created using LDlink [34], and for each SNP pair of strong linkage disequilibrium (*R*^2^ > 0.8, based on the CEU population from the 1000 Genome Project, phase 3 [35]) the SNP with the weaker myopia association measured by *P*-value was discarded. The final list consisted of 213 SNPs that are significantly associated with myopia among the Biobank participants at FDR = 0.05. We further confirmed at each of these SNPs that the reported risk allele is associated with a more negative refractive error. The effect size of the risk allele was estimated using the Biobank data.

### Allele frequency changes due to positive selection

Because deleterious alleles tend to be recessive, we considered beneficial alleles subject to positive selection to be dominant. Let *A* and *a* be two alleles at an SNP site, with allele frequencies of *p* and *q *= 1-*p*, respectively. Let the fitness of the genotype *aa* be 1 and those of *Aa* and *AA* be 1 + *s*, where *s* is the coefficient of selection. Using population genetic theory [36], one can show that the positive selection causes *p* to increase by Δ*p* ≈ *ps* per generation. Thus, *s* can be estimated by (Δ*p*)/*p*.

### Allele frequency changes across birth cohorts

At each SNP examined, we computed the frequency of the myopia-associated risk allele in each birth cohort using PLINK v2 [33]. We then tested the existence of a significant linear change in the allele frequency across the six cohorts at FDR < 0.05. We considered linear allele frequency changes because the formulation in the above section showed that, in a short period of time such as within a generation, the frequency of an allele subject to positive selection changes virtually linearly with a speed of *ps*/*g* per birth cohort, where *g* is the number of five-year birth cohorts per generation.

### Associations with lifespan and reproductive traits

Associations between SNPs and phenotypic traits were examined using PLINK v2 [33]. Specifically, to test the potential effect of an SNP on lifespan, we associated the genotype of a Biobank participant with the lifespan (only when available) of the participant's mother and that of his/her father, respectively. To test the potential effect of a SNP on reproduction, we associated the genotype of a Biobank participant with his/her AFB and NEB, respectively. Age, sex and the first 10 genetic principal components provided by the Biobank were fitted as covariates to avoid spurious associations.

### Local ancestry considered in association analysis

The local ancestry surrounding each of the 471 tested SNPs was estimated using LAMP-LD [37]. Specifically, the HapMap phase 3 [38] haplotype panels from CEU individuals with European ancestries and YRI individuals with African ancestries served as our reference populations to determine the parameters for the Hidden Markov Model (HMM). The local ancestry was then estimated using the HMM parameters within a 300-SNP sliding window and was coded by the number of alleles of European ancestries at each SNP at the individual level (i.e. 0, 1 or 2 standing for the number of alleles of European ancestries).

### Prediction of myopia phenotypes based on genotypes

We used the Genome-wide Complex Trait Analysis (GCTA) software [39] to predict the myopia phenotypes of 63 185 Biobank participants from their genotypes. The prediction was based on the following formula under an additive genetic model [39]:

$\hspace*{-18pt}\ {y_j} = \mathop \sum \nolimits_i {w_{ij}} {u_i} + {\varepsilon _j}, {{\rm {where}}\,\, {w_{ij}}}\, =\, ( {{x_{ij}}- 2{p_{i}}} )/$ $\sqrt {2{p_i}( {1 - {p_i}} )} $ . Here,$\ {y_j}$ is the phenotypic score of the *j*th individual, ${x_{ij}}$ is the number of reference alleles for the *i*th SNP of the *j*th individual, ${p_i}$ is the reference allele frequency of the *i*th SNP, ${u_i}$ is the allelic effect of the *i*th SNP, and ${\varepsilon _j}$ is the residual effect generated from a normal distribution with mean of 0 and variance equal to the empirical variance of $\mathop \sum \nolimits_i {w_{ij}}{u_i}$ multiplied by $( {\frac{1}{\scriptsize\textit{heritability}} - 1} )$. The myopia heritability was set at 35%. After excluding 21 SNPs with lifespan effects, we included 192 myopia-associated SNPs in the above formula. For the birth cohort of 1940–1944, a threshold (${y_0}$) was used such that the proportion of individuals with ${y_j} > {y_0}$ equals the observed myopia prevalence (30.3%) of the cohort. This same threshold was used for calling myopia from$\ {y_j}$ for any individual from any cohort, and the myopia prevalence was estimated for each cohort. The process was repeated 100 times and the averages were reported.

## Supplementary Material

nwaa175_Supplemental_File

## Data Availability

The data used were acquired from the UK Biobank (http://www.ukbiobank.ac.uk/about-biobank-uk/) under the license of Project 48678.
