# Supplementary material for: Natural selection contributes to the myopia epidemic
Source: Natl Sci Rev. Author manuscript; Available in PMC 2021 Sep 15. (PMC8288186; doi:10.1093/nsr/nwaa175)
Supplement: supplemental [file NIHMS1733597-supplement-supplemental.pdf]

Supplementary Materials for

Natural selection contributes to the myopia epidemic

E. Long & J. Zhang (jianzhi@umich.edu)

The supplementary materials include:
Supplementary Figures 1-3
Supplementary Table 1

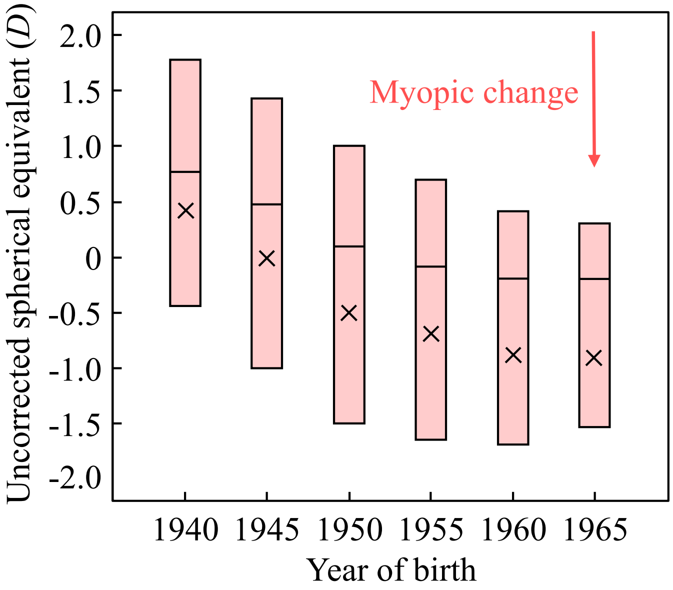

Figure S1. Uncorrected spherical equivalent (SpE) in six 5-year birth cohorts. The cross symbol represents the mean, the band shows the median, and the box indicates the middle 50% of individuals.

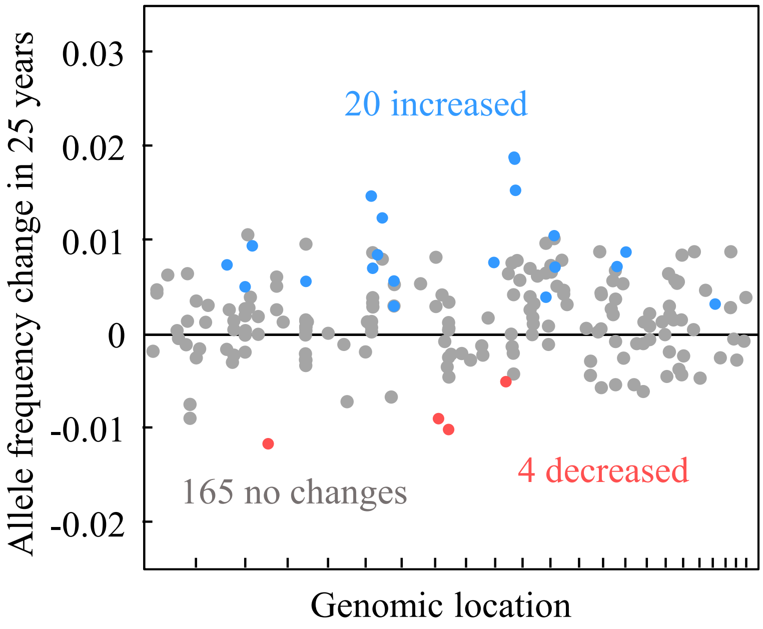

Figure S2. More myopia-associated alleles increased than decreased in frequency when the association study included the assessment center, genotyping batch, and local ancestry as additional covariates. Each dot represents one SNP, whose genomic coordinate is shown on the X-axis (chromosome 1 to 22 for each interval from left to right). Blue and red dots indicate SNPs with significant frequency changes, whereas grey dots indicate those without significant changes. The horizontal line indicates no frequency change.

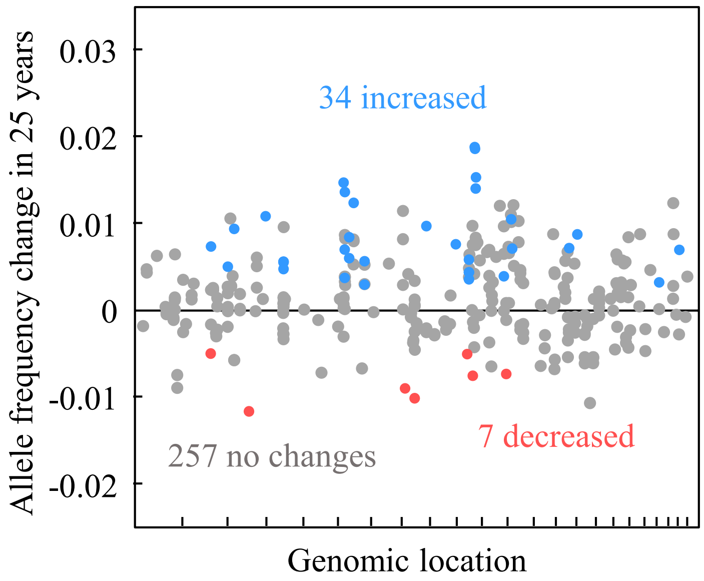

Figure S3. More myopia-associated alleles increased than decreased in frequency among all SNPs previously reported to be associated with myopia-related traits in other samples (after the removal of those with high linkage disequilibrium). Each dot represents one SNP, whose genomic coordinate is shown on the X-axis (chromosome 1 to 22 for each interval from left to right). Blue and red dots indicate SNPs with significant frequency changes, whereas grey dots indicate those without significant changes. The horizontal line indicates no frequency change.

Table S1. Myopia-associated risk alleles with significant frequency changes over the six birth cohorts but no significant effects on parent lifespan

Chromo -some	Position	SNP ID	Risk allele	Alternate allele	Risk allele frequency changes in 25 years	Effect on AFB in all data	Effect on NEB in all data	Effect on AFB in myopic individuals	Effect on NEB in myopic individuals	Effect on AFB in non-myopic individuals	Effect on NEB in non-myopic individuals
2	144466053	rs13382811	T	C	-0.0050	0.017	-0.0131	0.015	-0.0097	0.0262	-0.01
2	146131140	rs61049169	G	A	0.0074	-0.0311	0.0162*	-0.0225	0.0205*	-0.0221	0.0235*
2	233300046	rs2573232	T	C	0.0051	-0.096	0.0255*	-0.0687	0.0172*	-0.0405	0.0243*
3	24256698	rs4260345	C	T	0.0094	-0.0869*	0.0044	-0.0891	0.005	-0.1133*	0.0064
3	100125449	rs9811920	A	G	-0.0116	-0.007	-0.0123*	-0.0029	-0.0085	-0.0048	-0.0149*
3	186748463	rs1656966	G	A	0.0109	0.0196	-0.0011	0.0326	0.0006	0.0143	0.0004
4	81923677	rs10003846	T	G	0.0056	-0.0911	-0.0073	-0.0861	-0.0047	-0.0479	-0.0021
6	21160689	rs9295499	C	A	0.0147	-0.1075*	0.0108	-0.1136*	0.0245	-0.1129*	0.01
6	27195677	rs35909544	A	G	0.0070	0.0036	0.0035	0.0032	0.0061	0.0038	0.0083
6	27752933	rs36042294	G	C	0.0137	0.0566	-0.0046	0.0673	-0.0033	0.0448	0.0006
6	50757699	rs9395623	T	A	0.0085	-0.0925*	-0.0046	-0.0863	-0.0041	-0.0781	-0.004
6	50842007	rs2207136	T	C	0.0060	-0.1011*	-0.0114	-0.1116*	-0.0091	-0.1374*	-0.0094
6	73643289	rs7744813	A	C	0.0124	0.0051	-0.0069	0.0088	-0.0098	0.0057	-0.005
6	129513484	rs12205363	T	C	0.0030	0.0211	-0.0014	0.0167	-0.0025	0.0153	-0.0019
6	129842188	rs2326823	C	G	0.0057	0.0571	0.0045	0.0413	0.0035	0.0572	0.003
8	59219635	rs10113215	A	G	-0.0101	0.1117*	0.0043	0.1361*	0.0038	0.1194*	0.0048
8	120550178	rs7839488	G	A	0.0098	0.0282	0.0161*	0.0176	0.0211*	0.0088	0.0286*
9	129206832	rs10122788	G	A	0.0077	-0.0118	0.0019	-0.014	0.0091	-0.0088	0.0042
10	49414181	rs11101263	T	C	-0.0050	0.0844	-0.0146*	0.0607	-0.0116*	0.0505	-0.0152*
10	77303784	rs10824518	A	T	-0.0075	0.0362	0.007	0.046	0.0079	0.049	0.0077
10	88295142	rs11202704	T	C	0.0189	-0.0082	-0.0076	-0.0078	-0.0048	-0.0099	-0.0085
10	90142203	rs11202736	A	T	0.0187	-0.0084	-0.0064	-0.0053	-0.0046	-0.0073	-0.0025
10	93200318	rs55684140	G	T	0.0141	-0.0558	-0.0058	-0.0436	-0.0056	-0.0358	-0.0044
11	105642334	rs3170	A	T	0.0040	0.0271	0.0137*	0.0175	0.0119	0.0089	0.0094
12	9313304	rs7968679	G	A	0.0105	0.0504	-0.0045	0.0805	-0.0039	0.0685	-0.0053
12	14062637	rs4764038	T	G	0.0072	-0.0378	0.0061	-0.0419	0.0046	-0.0355	0.0038
14	60848527	rs1313240	C	T	0.0072	-0.1327*	0.0148*	-0.1523*	0.0092	-0.1485*	0.0051
14	104407243	rs35337422	C	A	0.0088	-0.1204*	-0.01	-0.1191*	-0.0087	-0.1093*	-0.0057
20	6761765	rs235770	T	C	0.0033	-0.0856*	-0.0089	-0.0799	-0.0113	-0.0901	-0.012
21	47377296	rs2150458	G	A	0.0070	-0.1186*	-0.0011	-0.119*	-0.0016	-0.1106*	-0.0022

AFB, age at first birth; NEB, number of children ever born, *, FDR < 0.05.
